# Nimotuzumab increases the anti-tumor effect of photodynamic therapy in an oral tumor model

**DOI:** 10.18632/oncotarget.3622

**Published:** 2015-04-20

**Authors:** Ramaswamy Bhuvaneswari, Qin Feng Ng, Patricia S.P. Thong, Khee-Chee Soo

**Affiliations:** ^1^ National Cancer Centre Singapore, Division of Medical Sciences, Singapore 169610, Singapore; ^2^ Duke-NUS Graduate Medical School, Singapore 169857, Singapore

**Keywords:** photodynamic therapy, epidermal growth factor receptor, nimotuzumab, oral squamous cell carcinoma, angiogenesis

## Abstract

Oral squamous cell carcinoma (OSCC) represents 90% of all oral cancers and is characterized with poor prognosis and low survival rate. Epidermal growth factor receptor (EGFR) is highly expressed in oral cancer and is a target for cancer therapy and prevention. In this present work, we evaluate the efficacy of photodynamic therapy (PDT) in combination with an EGFR inhibitor, nimotuzumab in oral cancer cell lines and OSCC xenograft tumor model. PDT is a promising and minimally invasive treatment modality that involves the interaction of a photosensitizer, molecular oxygen and light to destroy tumors. We demonstrated that EGFR inhibitors nimotuzumab and cetuximab exhibits anti-angiogenic properties by inhibiting the migration and invasion of oral cancer cell lines and human endothelial cells. The EGFR inhibitors also significantly reduced tube formation of endothelial cells. Chlorin e6-PDT in combination with nimotuzumab and cetuximab reduced cell proliferation in different oral cancer and endothelial cells. Furthermore, our *in vivo* studies showed that the combination therapy of PDT and nimotuzumab synergistically delayed tumor growth when compared with control and PDT treated tumors. Downregulation of EGFR, Ki-67 and CD31 was observed in the tumors treated with combination therapy. Analysis of the liver and kidney function markers showed no treatment related toxicity. In conclusion, PDT outcome of oral cancer can be improved when combined with EGFR inhibitor nimotuzumab.

## INTRODUCTION

Oral squamous cell carcinoma (OSCC) is not only the sixth most prevalent malignancy worldwide but also has the poorest clinical outcomes [1]. Conventional therapies such as chemotherapy, radiation and surgery are effective but side effects can be significant. In spite of advances in various treatment modalities, oral cancer is still a disease of high morbidity and mortality [2]. Therefore there is a need for targeted therapy to minimize side effects and to improve selective destruction of cancer cells. The epidermal growth factor receptor (EGFR) has been implicated in OSCC carcinogenesis and is overexpressed in up to 90% of head and neck squamous cell carcinoma (HNSCC) [3, 4]. Overexpression of EGFR is associated with aggressive behaviour including increased proliferation, metastasis, and therapeutic resistance in squamous cell carcinoma (SCC) of the oral cavity and oropharynx [5]. Therefore, since increased expression of EGFR is associated with poor clinical outcome, that makes it a promising and attractive therapeutic target [6]. In this study we investigate a targeted approach by treating oral cancer with an anti-EGFR monoclonal antibody in combination with photodynamic therapy (PDT).

There is significant interest in the use of PDT as an alternative therapeutic modality to treat early head and neck cancer. As oral cancer is highly aggressive and recurrent in nature, PDT holds promise as it can be safely repeated with no cumulative toxicity. It is minimally invasive with short term side effects that include pain especially during treatment, swelling and photosensitivity [7]. Chlorin e6 (Ce6), a second-generation photosensitizer was used to perform the PDT experiments. Ce6 has a high quantum yield with a singlet oxygen production of 0.65 [8] and its amphiphilic structure allows easy penetration through cell membrane, thus ensuring effective uptake within cells [9]. PDT is a clinically approved minimally invasive modality that involves the selective uptake of the photosensitizer in the tumor tissue which is then irradiated with light of specific wavelength. This interaction between the drug and light causes the production of reactive oxygen species that result in tumor cell destruction [10]. The results from a phase I/II trials conducted on head and neck tumors strongly suggest that PDT could be an effective primary and alternative treatment modality for patients presenting with early head and neck tumors [11]. In another study, a total of 30 regions in 25 patients (18 with squamous cell carcinoma and 7 with epithelial dysplasia with hyperkeratosis in the oral cavity) were treated by PDT. Complete response was achieved in 24 of the 25 patients (96%), with a partial response found in the remaining patient [12].

Many studies have reported the major role of EGFR in PDT response [13]. It has been noted that the ERK1/2 and EGFR-PI3K-Akt pathways could be involved in cellular survival after PDT. A recent study reported that BPD-mediated PDT initiates nuclear signaling of EGFR and STAT3 which results in decreased cancer cell cytotoxicity following PDT. This suggests that EGFR inhibitors can have potential synergistic effect when administered with PDT, and this could be highly relevant for clinical use [14]. In fact, numerous studies have demonstrated that PDT and EGFR inhibitors in combination act synergistically *in vitro* and *in vivo* [15–19]. We have previously reported that combined therapy with PDT and the EGFR inhibitor cetuximab inhibited tumor growth in a bladder human cancer model [20]. In this study we use nimotuzumab (also mentioned as nimo in the figures) which is a humanized IgG_1_ monoclonal antibody that binds to the extracellular domain of the EGFR, thus inhibiting EGF binding. It also has unique functional properties compared to other anti-EGFR antibodies [21]. It selectively binds to cells that express moderate to high EGFR levels, as it intrinsically requires bivalent binding for stable attachment to the cellular surface. As nimotuzumab has lesser affinity to low EGFR expressing cells, it spares healthy tissues and avoids the severe dose limiting toxicities seen in other anti-EGFR monoclonal antibodies [22].

Nimotuzumab has shown potent antiproliferative, antiangiogenic and proapoptotic activity in A431 squamous cell carcinoma cells [23]. In patients with HNSCC, nimotuzumab treatment can lead to long-term stable disease with a low toxicity profile, in contrast to other anti-EGFR agents [24–26]. Nimotuzumab in combination with irradiation or chemoradiation was safe and tolerable for patients with SCC of the esophagus, and yielded encouraging overall survival, progression free survival and locoregional control [27]. In this study, the combination of PDT and nimotuzumab has shown anti-cancer properties by decreasing angiogenesis, increasing apoptosis and by delaying tumor growth in an oral cancer tumor model.

## RESULTS

### OSCC, HSC-3 and SCC-25 cells overexpresses EGFR

**Figure 1:**
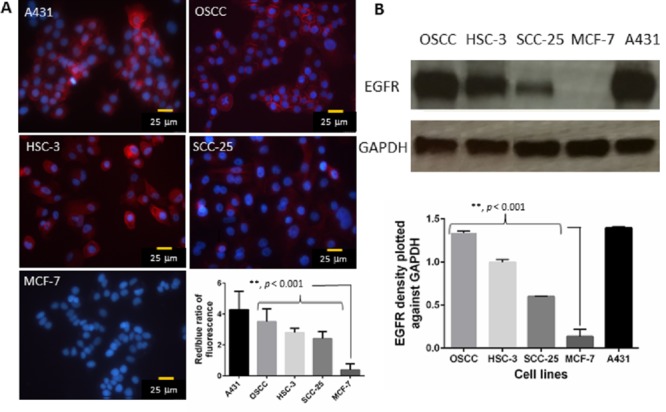
A. Representative immunofluorescence images show the expression of EGFR in A431 (positive control), OSCC, HSC-3, SCC-25 and MCF-7 cells (negative control) Red fluorescence represents EGFR and the blue fluorescence depicts nuclei, stained by Hoechst 33342. **B.** Western blotting analysis was performed to confirm the above results. The ratio of EGFR intensity was plotted against GAPDH. Error bars represents standard error of the mean.

Immunofluorescence assay was performed to assess the expression of EGFR in OSCC, HSC-3 and SCC-25 cells (Figure 1A). An epidermoid carcinoma cell line (A431) was used as the positive control as these cells are known to overexpress EGFR. MCF-7, a breast cancer cell line that expresses low levels of EGFR, served as a negative control. In the immunofluorescence study, Hoechst 33342 was used to stain the nucleus (blue). Secondary antibody tagged with Texas red was used to detect EGFR. Image analysis was performed by quantifying the red and blue intensities of the images and the red to blue fluorescence ratio was calculated. Our results showed highly significant difference (*p* < 0.001) in the expression of EGFR in OSCC (3.5), HSC-3 (2.8) and SCC-25 (2.4) cells compared to MCF-7 (0.4) cells. Significantly higher red to blue ratio was observed for OSCC and HSC-3 cells compared to SCC-25 cells. Expression of EGFR in all the cell lines was reconfirmed using Western blotting (Figure 1B). The ratio of EGFR intensity plotted against GAPDH was highest for OSCC (1.3) compared to HSC-3 (0.9) and SCC-25 cells (0.6).

### Nimotuzumab and cetuximab exhibit anti-angiogenic properties

**Figure 2:**
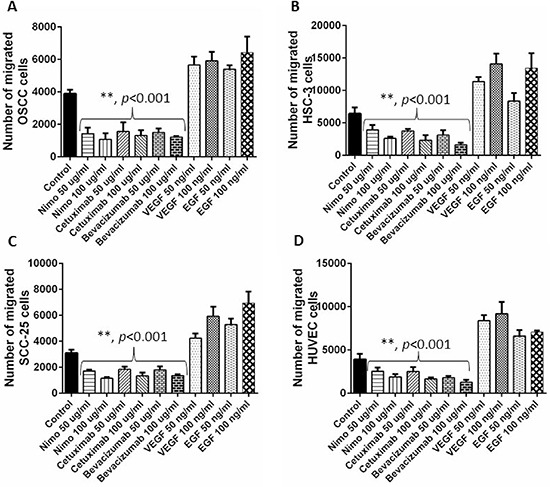
Cell migration assay was performed to understand the anti-angiogenic effects of EGFR inhibitors, nimotuzumab and cetuximab in OSCC A. HSC-3 B., SCC-25 C. and HUVEC D. cells Migrated cell number was determined by performing a fluorescent cell dose curve. Bevacizumab was used as the positive control and VEGF and EGF were used as negative controls. Error bars represent the standard error of the mean, *n* = 6 wells.

**Figure 3:**
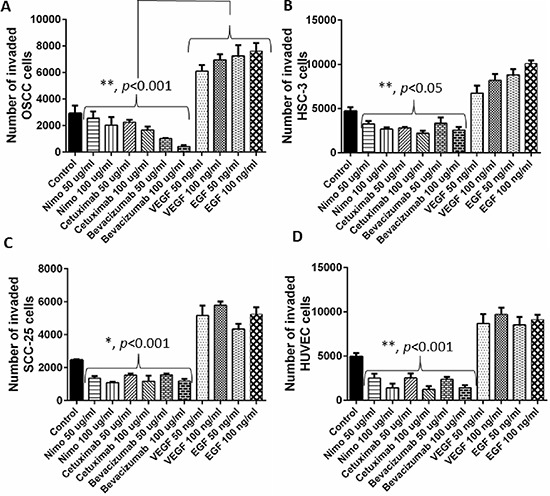
Invasion of OSCC. A. HSC-3 B. SCC-25 C. and HUVEC D cells through the extracellular matrix (ECM) was assessed understand the anti-angiogenic effects of EGFR inhibitors, nimotuzumab and cetuximab. Number of invaded cells was determined by performing a fluorescent cell dose curve. Bevacizumab acted as the positive control and VEGF and EGF were used as negative controls. Error bars represent the standard error of the mean, *n* = 6 wells.

**Figure 4:**
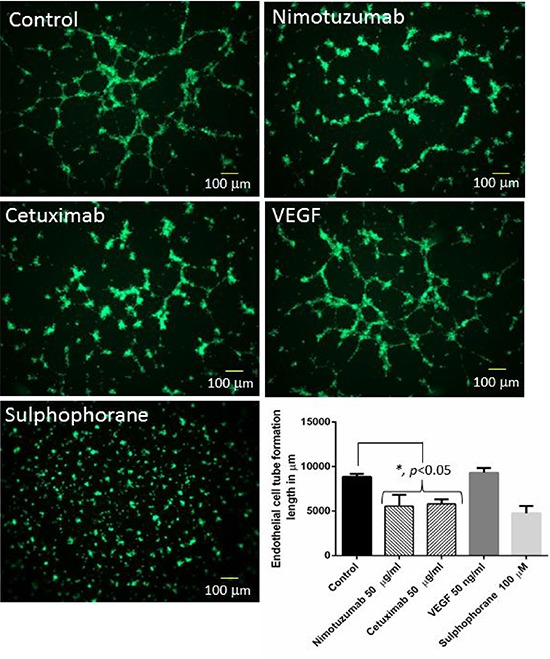
Representative images of tube formation using HUVEC cells Nimotuzumab and cetuximab disrupted tube formation compared to control. VEGF was used as the positive control and sulphophorane was used as the negative control. The length of endothelial tube formation was measured in μm using Image Pro Plus 6.0 software.

Cell migration, invasion and tube formation assays were performed to investigate the anti-angiogenic properties of nimotuzumab and cetuximab. For cell migration and invasion assays, bevacizumab was used as the negative control as it is a known anti-angiogenic agent and VEGF and EGF were used as the positive controls as they are known to promote angiogenesis. Cetuximab is a humanized antibody that is directed at the extracellular domain of the EGFR, preventing ligand binding and thus promoting the activation of the receptor. This blocks the downstream signaling of EGFR resulting in impaired cell growth and proliferation. Cell migration is a fundamental activity intrinsic for tumor growth and development. Understanding the process of cell migration to different sites is important in order to block and prevent tumor progression. Migration of cells in all the four cell lines was comparable. Cell migration was significantly lower in nimotuzumab, cetuximab and bevacizumab treated cells compared to control (*p* < 0.001). Almost 2 to 3 fold increase in migration was observed in the cells treated with proangiogenic factors, VEGF and EGF. At higher concentration of 100 μg/ml lower number of cells migrated compared to 50 μg/ml, however this difference was not significant for all the cell lines. No significant difference in migration was observed between cells treated with nimotuzumab, cetuximab and bevacizumab (Figure 2). Similar trend was observed in the invasion of the cells through extracellular matrix (Figure 3). Invasion of cells was significantly lower for all the inhibitor groups compared to control in all the cell lines except OSCC. Endothelial cells need to cross the basement membranes in order to disseminate or to form new blood vessel, therefore it is important to understand the effect of the EGFR inhibitors on the invasion of endothelial barriers. In the endothelial tube formation assay, endothelial cells were plated on a gelled basement matrigel to form capillary-like structures with a lumen. Pro-angiogenic agents are known to promote tube formation and anti-angiogenic agents are known to inhibit the formation of endothelial tubes. For this assay, VEGF was used as the positive control as it is known to promote angiogenesis and sulphophorane was used as the negative control as it disrupts tube formation. Nimotuzumab and cetuximab disrupted tube formation of endothelial cells and was comparable to the negative control, sulphophorane. With nimotuzumab and cetuximab the average length of endothelial tubes was significantly lower at 5550 μm and 5802 μm (*p* < 0.05) compared to control and VEGF groups. For the control and VEGF treated endothelial cells, increased tube formation lengths of approximately 8800 μm and 9300 μm were observed respectively (Figure 4).

### PDT and EGFR inhibitors reduce proliferation of different oral cancer cells and HUVECs

**Figure 5:**
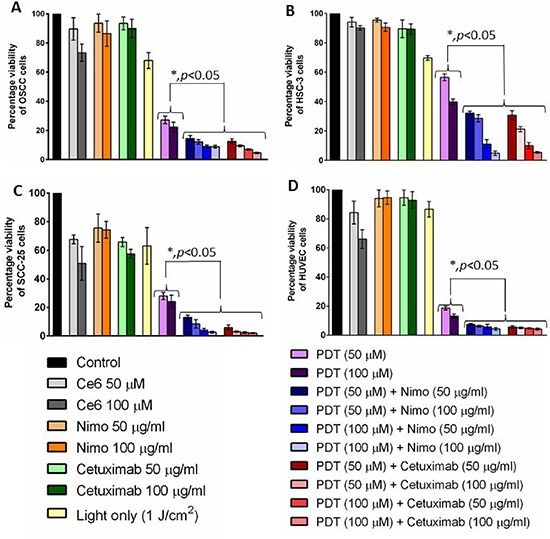
Cell viability was measured quantitatively using a colorimetric detection kit in OSCC. A. HSC-3 B. SCC-25 C. and HUVEC D for different treatment groups. Percentage cell viability was calculated against control. Combination of PDT and EGFR inhibitors, nimotuzumab and cetuximab significantly (*p* < 0.05) increased tumor cell death in all the cell lines compared to the PDT treatment alone. Error bars represent the standard error of the mean of triplicate experiments.

Both PDT and the combination of PDT and nimotuzumab significantly increased tumor cell death in all the cell lines. Treatment response in OSCC (Figure 5A) and SCC (Figure 5C) cells was comparable with 22 to 27% (*p* < 0.001) cell survival post PDT and significantly lower cell viability of 2–14% (*p* < 0.001) in the combination therapy groups of PDT + nimotuzumab and PDT + cetuximab compared to control. In comparison lower cell death was observed in HSC-3 cells (Figure 5B). 40–56% cell survival was observed post PDT and variable range of 4.8 to 32% cell viability was observed in the combination therapy groups of PDT + EGFR inhibitors compared to control. We also observed that in HSC-3 cells the higher dose of PDT (100 μM) + EGFR inhibitor (100 μg/ml) induced significantly greater cell death compared to lower dose of PDT (50 μM) + EGFR inhibitor (50 μg/ml). Surprisingly, HUVEC cells responded well to the PDT treatment (Figure 5D). Around 13 to 19% (*p* < 0.001) cell survival was observed post PDT and significantly lower cell viability of 4 to 7.5% (*p* < 0.001) was observed in the combination therapy groups compared to control. In the control groups, for all cell lines, cell viability of 63 to 86% was observed with light treatment only and 74 to 95% cells were observed when treated with EGFR inhibitors only compared to control.

### PDT and EGFR inhibitors acts synergistically on oral cancer cells

For 2 different combinations of PDT and EGFR inhibitors treatment (PDT-50 μM + inhibitor-50 μg/ml) and (PDT-100 μM + inhibitor-100 μg/ml), the synergistic/additive or antagonistic effects was statistically calculated for all the 4 cells lines. Based on the difference in logarithms (DL) and *p* values, it was determined that PDT + nimotuzumab and PDT + cetuximab synergistically induced cell death in all the cell lines (Table 1).

**Table 1:** The difference in logarithm (DL) values, Standard error (SE) of DL values, *p* values based on *t*-test and the synergistic/additive or antagonistic effect of the different treatment combinations in all the four cell lines and *in vivo* combination studies have been listed

Cell lines	Treatment	DL value (mean)	Standard Error (SE) of DL	*p* value	Synergistic/additive or antagonistic
OSCC	PDT (50 μM) + Nimo (50 μg/ml)	0.2348	0.03336	0.0009	Synergistic
OSCC	PDT (100 μM) + Nimo (100 μg/ml)	0.2882	0.03500	0.0004	Synergistic
OSCC	PDT (50 μM) + Cetuximab (50 μg/ml)	0.3003	0.01797	< 0.0001	Synergistic
OSCC	PDT (100 μM) + Cetuximab (100 μg/ml)	0.5812	0.03609	< 0.0001	Synergistic
HSC-3	PDT (50 μM) + Nimo (50 μg/ml)	0.5535	0.01455	< 0.0001	Synergistic
HSC-3	PDT (100 μM) + Nimo (100 μg/ml)	0.5865	0.2002	0.0428	Synergistic
HSC-3	PDT (50 μM) + Cetuximab (50 μg/ml)	0.7602	0.1864	0.0151	Synergistic
HSC-3	PDT (100 μM) + Cetuximab (100 μg/ml)	0.4358	0.04715	0.0008	Synergistic
SCC-25	PDT (50 μM) + Nimo (50 μg/ml)	0.3321	0.05054	0.0012	Synergistic
SCC-25	PDT (100 μM) + Nimo (100 μg/ml)	1.475	0.1226	< 0.0001	Synergistic
SCC-25	PDT (50 μM) + Cetuximab (50 μg/ml)	1.187	0.3539	0.0202	Synergistic
SCC-25	PDT (100 μM) + Cetuximab (100 μg/ml)	1.663	0.03313	< 0.0001	Synergistic
HUVEC	PDT (50 μM) + Nimo (50 μg/ml)	0.3881	< 0.0001	< 0.0001	Synergistic
HUVEC	PDT (100 μM) + Nimo (100 μg/ml)	0.3240	0.0136	0.0136	Synergistic
HUVEC	PDT (50 μM) + Cetuximab (50 μg/ml)	0.5184	< 0.0001	< 0.0001	Synergistic
HUVEC	PDT (100 μM) + Cetuximab (100 μg/ml)	0.4599	< 0.0001	< 0.0001	Synergistic
*In-vivo* PDT with OSCC cells	PDT (10 mg/kg) + Nimo (10 mg/kg)	0.4840	0.1290	0.0199	Synergistic

### PDT and nimotuzumab delays *in-vivo* tumor growth and survival rate

**Figure 6:**
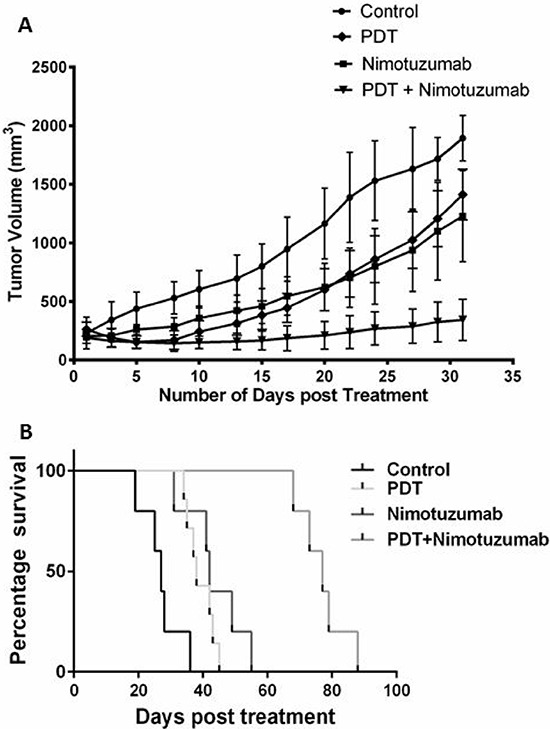
Tumor volume was charted against days, to assess the tumor response in various treatment groups for 32 days **A.** Tumors treated with the combination therapy of PDT + nimotuzumab exhibited significantly greater (*p* < 0.05) treatment response compared to control, nimotuzumab and PDT treated groups. Each group represents the mean (bars, SE) of 10 animals. Kaplan–Meier survival curve was also plotted for various treatment groups for 90 days **B.**

Based on the *in vitro* results we decided to use OSCC cells to develop the tumor xenograft model in athymic nude mice to investigate the long-term effectiveness of PDT and nimotuzumab. Tumors were allowed to grow to sizes of 6–7 mm in diameter before PDT treatment was carried out and tumor were measured three times a week for a 90-day period (Figure 6A). For tumor regression experiments, animals were sacrificed at day 32 when control tumors reached the maximum tumor limit. Tumors treated with the combination therapy of PDT + Nimotuzumab exhibited significantly greater treatment response compared to control, nimotuzumab and PDT treated groups (*p* < 0.05). No significant difference in the tumor growth of PDT alone and nimotuzumab alone mono-therapy groups was observed. We also looked at the percentage survival for the different treatment groups. Kaplan–Meier survival curve was plotted for various treatment groups for 90 days (Figure 6B). The percentage survival of 88% was observed in animals treated with PDT + nimotuzumab which was significantly greater compared to control (36%), PDT alone (45%) and nimotuzumab alone (55%) treated animals. Also we statistically determined that PDT + nimotuzumab synergistically inhibits tumor growth (*p* < 0.0199) (Table 1). The treatment modalities in this study did not induce any signs of toxicity such as excessive weight loss, diarrhoea or vomiting in the animals. No treatment-related death occurred.

### Assessment of EGFR, Ki-67 and CD31 expression in tumor tissue

**Figure 7:**
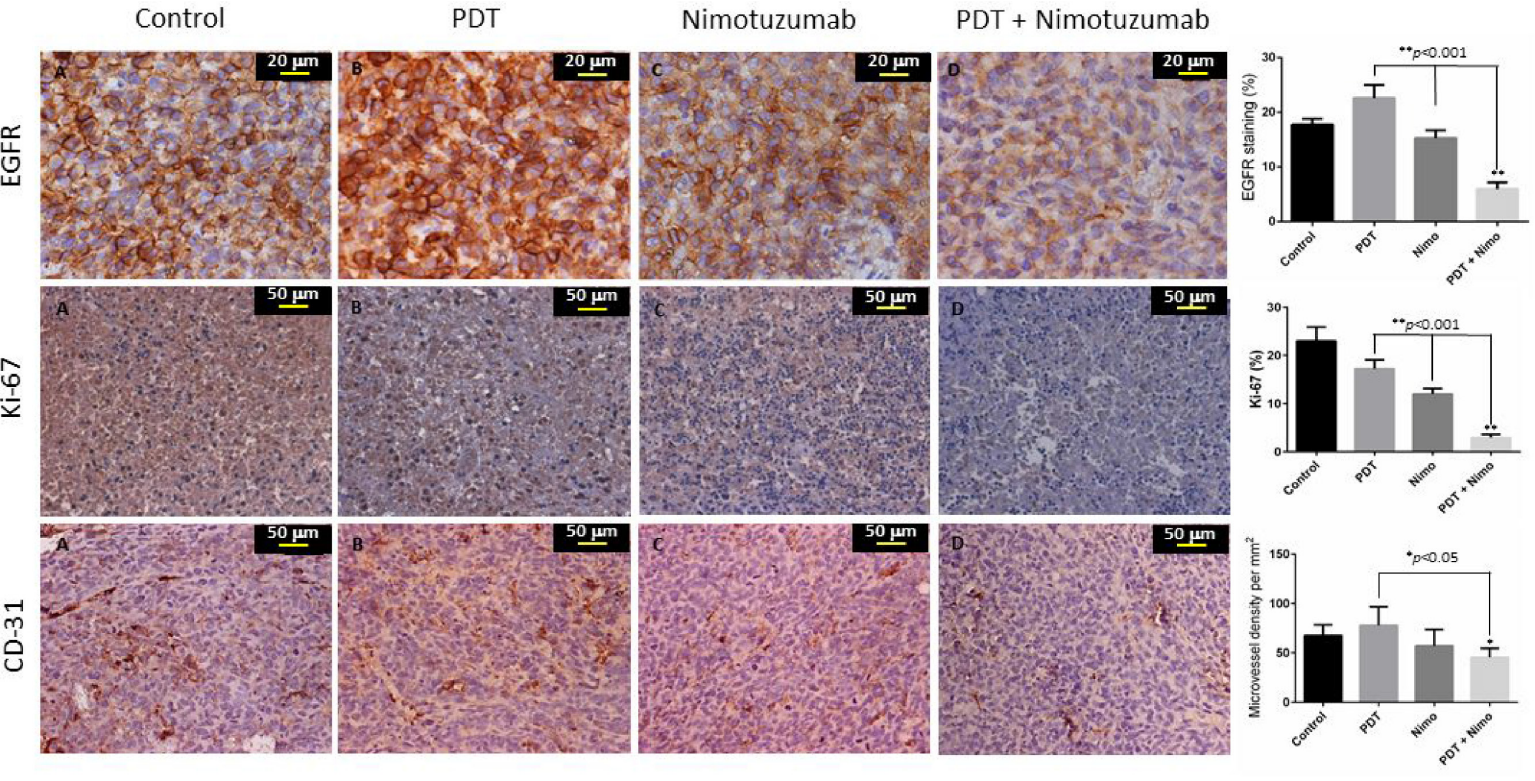
Representative IHC images of paraffin embedded tumorsections post treatment Percentage of EGFR and Ki-67 staining and microvessel density per mm^3^ measurement of CD31 was determined. EGFR staining was observed in the cell member and Ki-67 stained the nuclei of proliferating cells. CD31 staining showed vessel damage in treated tumors compared to control. Open lumen vasculature was observed in the combination therapy treated tumors.

Immunohistochemistry was performed on paraffin embedded tumor tissue for all the 4 treatment groups (Figure 7). EGFR expression was evaluated in the various treatment groups. As EGFR is a membrane protein, staining of the cell membrane was observed. Increased EGFR expression was observed in the tumors treated with PDT (22.6%) when compared to control (17.8%) and nimotuzumab treated tumors (15.3%). Mild EGFR staining (6%) was observed in the combination therapy group of PDT + nimotuzumab. The nuclear antigen Ki-67 is a proliferation marker expressed only in cycling cells. Ki-67 staining was significantly lower (*p* < 0.001) in the PDT + nimotuzumab treated tumors (3%) compared to control (23%) and tumors treated with PDT only (17.2) and nimotuzumab only (12). Cluster of differentiation (CD31) staining was performed to assess angiogenesis, which also predicts tumor recurrence. The endothelial cells stained by CD31 in the tumor tissue were not well organized and the vessels were fragmented compared to normal blood vessels. Significantly lower (*p* < 0.001) and more scattered microvessel staining (CD31) was observed in the PDT + nimotuzumab (Microvessel density (MVD) 45.4/mm^2^) tumors compared to control (MVD 67.4/mm^2^), PDT (MVD 77.8/mm^2^) and nimotuzumab treated tumors (MVD 57/mm^2^). However, open lumen vasculature were observed in the combination therapy treated tumors which may suggest vessel maturation and increased functionality of vessels.

### Toxicity studies

**Figure 8:**
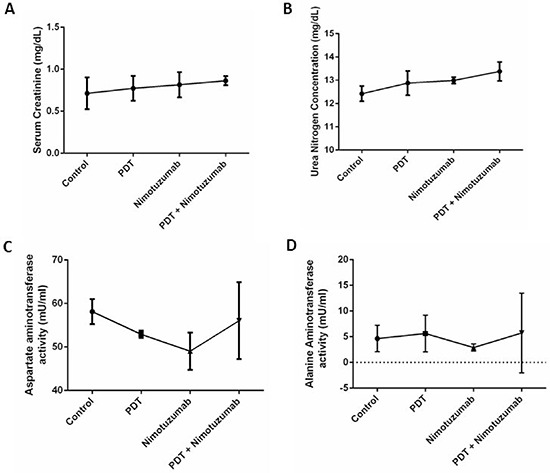
Toxicity studies were performed by measuring serum creatinine. A. urea nitrogen B. aspartate aminotransferase C. and alanine aminotransferase levels D in serum to understand the kidney and liver function in mice treated with different therapies. The levels of all the enzymes were within the normal range. Error bars represent the standard error of the mean of triplicate experiments.

Toxicity studies were performed by measuring creatinine, urea nitrogen, aspartate aminotransferase (AST) and alanine aminotransferase (ALT) levels in mouse serum to understand the kidney and liver function following different therapies. The range for serum creatinine was 0.5 to 0.9 mg/dL in all the groups which was within the normal range (0.1–2.1 mg/dL) (Figure 8A). The range for urea nitrogen was 12 to 13.5 ug/ml in the different groups which was within the normal range (2–71 mg/dL) (Figure 8B). The urea nitrogen mildly increased in the PDT + nimotuzumab (13.5 mg/dL) group compared to control (12.4 mg/dL), PDT (12.8 mg/dL) and nimotuzumab (13 mg/dL) treated animals. However, there were no significant differences among the four groups. AST levels were around 44–58 mU/ml which is within the normal range of 37–329 mU/ml (Figure 8C). ALT levels were around 0.5 to 17 mU/ml but the normal range is 7–227 mU/ml (Figure 8D). Some serum samples from animals had ALT levels below the normal range. Surprisingly a dip in aspartate aminotransferase (48 mU/ml) and alanine aminotransferase (3 mU/ml) was observed in the animals that were treated with nimotuzumab alone. However, no significant difference was observed in AST and ALT levels between the various groups.

## DISCUSSION

Photodynamic therapy (PDT) is an effective adjuvant to standard conventional treatments for the management of head and neck neoplasms. Important advantages of PDT are that it is a localized treatment and can be repeated as often as required, has low systemic toxicity, provides excellent cosmetic outcome and lessens morbidity. Despite its success, tumor recurrence has been reported in squamous cell carcinoma (SCC) patients [29]. This could be due to certain limitations of PDT that include lack of penetration depth of the activating light and incomplete treatment of the margins compared to the center of the tumor. The residual tumor cells can then repopulate and grow aggressively, leading to tumor recurrence. Recent studies suggest that overexpression of EGFR results in poor prognosis in oral cancer and its activation is associated with malignant phenotype, inhibition of apoptosis and increased metastatic potential [1]. Therefore, there is a need for targeted therapy to improve the efficacy of PDT. Here, we investigate the efficacy of EGFR inhibitor nimotuzumab in combination with PDT to effectively treat oral tumor xenografts *in vivo*.

In this study, we first estimated the expression of EGFR in OSCC, HSC-3 and SCC-25 cells. Since most oral cancers are epithelial in origin, there is a high probability of EGFR overexpression [6]. Both immunofluorescence and western blotting results showed a substantial amount of EGFR expression in oral cancer cells. However, expression of EGFR was greater in OSCC and HSC-3 compared to SCC-25 cells. Based on these results we decided to use the OSCC cell line to establish an oral cancer xenograft tumor model for the *in vivo* regression and survival studies. Overexpression of EGFR has been observed in up to 90% of head and neck squamous cell carcinomas. EGFR activation is known to be associated with the malignant phenotype, angiogenesis, inhibition of apoptosis as well as increased metastatic potential in oral cancer [30]. Therefore inhibition of EGFR and its downstream signalling pathways presents a promising strategy to prevent tumor progression.

Nimotuzumab is a potent EGFR inhibitor that requires bivalent binding for stable attachment and would therefore bind preferentially to tumor cells having a medium to high surface density of EGFR protein. For this reason, nimotuzumab exhibits a lower toxicity profile compared to cetuximab that can bind to normal cells with lower EGFR expression levels [31]. In this study, we were interested in understanding the anti-angiogenic effects of nimotuzumab on oral cancer and human endothelial cells. Therefore we performed cell migration, invasion and tube formation assays to assess the anti-angiogenic properties of nimotuzumab and compared it with cetuximab. Cetuximab is a chimeric human-murine monoclonal antibody that competitively binds to the accessible extracellular domain of EGFR and inhibits dimerisation and subsequently inhibits cell proliferation, tumor growth and metastasis [32]. Based on our results, nimotuzumab could successfully prevent endothelial cell migration, invasion and endothelial cell tube formation and moreover its effects were comparable to cetuximab, and bevacizumab which is a well-known anti-angiogenic agent. Two different concentrations of the inhibitors were tested and no significant difference in the migration or invasion of cells was observed though the concentration was doubled. This could be due to the fact that to begin with the concentration of inhibitors was high at 50 μg/ml, which already prevented substantial migration and invasion of cells so doubling the concentration did not make a difference. A 2–3 fold increase in migration and invasion of cells was observed when treated with VEGF and EGF proteins compared to control. Cell migration and invasion are crucial steps that can lead to tumor metastasis. In HNSCC, tumor cells attract monocytes and macrophages to activate the secretion of growth factors and cytokines that stimulate the production of interleukin-8 (IL-8) and vascular endothelial growth factor (VEGF) [23]. A recent study has reported that EGF-Like-Domain-7, a secreted angiogenic factor, is required for VEGF-Induced Akt/ERK activation and vascular tube formation [33]. Therefore, the role of nimotuzumab in the prevention of angiogenesis in addition to EGFR targeting would be an added advantage in controlling the invasion of head and neck squamous cell carcinoma. Both nimotuzumab and cetuximab significantly disrupted tube formation of endothelial cells. Earlier reports have shown that nimotuzumab might possess a VEGF-mediated anti-angiogenic component in their mechanism of action. This hypothesis was reinforced by a study that reported a significant 3-fold reduction in microvessel density [34], which occurs in parallel to the decrease in VEGF expression following nimotuzumab treatment. Likewise, a reduction in vessel size, which is often associated with an anti-angiogenic response, is also evident in nimotuzumab-treated tumors [35]. It is known that VEGF promotes the survival of endothelial cells by preventing apoptosis [35]. However, this effect can be reversed by administering nimotuzumab which increases apoptosis by reducing the production of VEGF, leading to reduced neovascularity. In line with our study, previous reports suggest that nimotuzumab is anti-angiogenic and could serve as a targeted therapy against both endothelial cells and tumor cells by disrupting the vascular endothelial microenvironment [36].

Next we performed *in vitro* experiments to evaluate cell viability post treatment on 3 different oral cell lines and HUVECs. Based on statistical calculations, it was noted that different dosages of both PDT + nimotuzumab and PDT + cetuximab synergistically induced cell death in all the cell lines. The fundamental rationale for combination therapy is to use approaches that work by different mechanisms of action. The combination effect can be synergistic, additive or in some cases antagonistic. Synergistic effects of different combinations of treatments can significantly enhance therapeutic outcome. In order to obtain synergistic effect, PDT can be combined with agents that can counteract prosurvival signalling triggered in tumor cells that can develop resistance to PDT [37]. Ce6-PDT significantly increased cell death in oral cancer cells and nimotuzumab and cetuximab significantly enhanced the anti-tumor effect of PDT. Similar to our results, pheophorbide-a (Pa)-PDT in an oral cancer cell line reduced cell viability by 50% and produced reactive oxygen species (ROS) [38]. Compared to this, Ce6-PDT reduced cell viability by around 70%. Ce6-PDT predominantly causes apoptosis of tumor cells and about 80% apoptosis was observed in colon carcinoma CT-26 when treated with a light dose of 1 J/cm^2^ [39]. However, the percentage of apoptosis can always differ based on the PDT dosage and type of tumor model used. Our preliminary results using flow cytometry has shown almost 50% apoptosis with combination therapy of PDT and nimotuzumab compared to control (10%) at 24 hour time-point (Supplementary Figure 2A and 2B). Photosensitization of human vascular smooth muscle cells with Ce6 has also shown to result in the generation of ROS, a decrease in cell membrane polarization, caspase-3 activation, as well as DNA-fragmentation [40]. Similar to our results, the efficacy of Zn-BC-AM-PDT was increased in nasopharyngeal carcinoma cells (NPC) through the inhibition of EGFR/PI3K/Akt and EGFR/MEK/ERK signaling pathways using an EGFR inhibitor AG1478 [41]. Both nimotuzumab and cetuximab by itself reduced cell viability by only 10–40% in various cell lines because even though it is highly tumor specific by itself it is low in toxicity. No significant difference in cell death was observed between the two cetuximab and nimotuzumab doses of 50 μg/ml and 100 μg/ml. Though the results for cetuximab and nimotuzumab are comparable, this study mainly focused on the use of nimotuzumab in our *in-vivo* experiments as it is known that nimotuzumab is less toxic than cetuximab as it needs to bivalently bind to EGFR molecules and therefore normal tissues that express lower levels of EGFR are not affected [22]. Nimotuzumab targets EGFR and blocks the binding of the ligand while permitting the conformation of the active receptor. It has been reported that this antibody can suppress cell proliferation in epithelial cancer by causing cell cycle arrest. When the antibody binds to the extracellular domain of EGFR, it can strongly inhibit EGFR-dependent cellular transformation. Similar to our results with PDT, nimotuzumab has shown to selectively enhance anti-tumor effects of ionizing radiation of non-small-cell lung carcinoma (NSCLC) cell lines with high EGFR expression [42]. Furthermore, combination of nimotuzumab with radiation increased apoptosis and G2/M phase arrest in human lung adenocarcinoma A549 cells [43]. Nimotuzumab also promoted radiosensitivity of esophageal squamous cell carcinoma cells by up-regulating IGFBP-3 through EGFR-dependent pathway [44]. Surprisingly, we noticed an enhanced effect of PDT and PDT combination therapy on HUVEC cells compared to other oral cancer cells. It has been widely reported that vascular-targeting PDT can induce alterations in the tumor vasculature leading to vasoconstriction, microvascular shutdown and blood flow stasis [45]. Vascular photosensitization can permeabilize blood vessels through the formation of endothelial intercellular gaps, which are likely induced via endothelial cell microtubule depolymerization. The loss of endothelial barrier function can ultimately lead to tumor vascular shutdown and will have significant implications in drug transport and tumor cell metastasis [46]. Thus, modifying the PDT protocol to target both the vasculature and cellular components in the tumor microenvironment can substantially improve treatment outcome.

In the *in vivo* tumor regression experiments, we compared the tumor growth between the various treatment groups. Nimotuzumab was used at a dosage of 10 mg/kg based on our optimization experiments whereby 2 mg/kg, 5mg/kg and 10mg/kg drug doses were tested with Ce6-PDT. 10 mg/kg nimotuzumab with PDT (150 J/cm^2^ and 100 mW/cm^2^) exhibited increased tumor inhibition compared to 2 mg/kg and 5 mg/kg nimotuzumab (Supplementary Figure 1). As we have used Ce6-PDT extensively in our previous studies, the drug-light interval was optimized at 3 hours [47, 48]. We monitored the tumors until 32 days as the control tumors had reached the maximum ethical limit by then. Our *in vivo* experiments showed that the combination therapy of PDT and nimotuzumab significantly reduced tumor growth compared to the monotherapy groups and control. The rate of tumor growth in the monotherapy groups, PDT alone and nimotuzumab alone, was comparable and lower than the rate in untreated control tumors. The Kaplan Meier survival curve that was charted for 90 days also showed increased percentage of survival of animals treated with the combination therapy compared to monotherapies alone. In an earlier study we have also demonstrated that targeting EGFR using cetuximab improved the efficacy of PDT treatment in a human bladder cancer model. Increased apoptosis and downregulation of EGFR target genes cyclin D1 and c-myc was observed in tumors treated with PDT and cetuximab [20]. Inhibition of EGFR signalling can lead to increased PDT cytotoxicity through a mechanism that involves increased apoptotic cell death [14]. Similarly, PDT + C225, an EGFR inhibitor, produced synergistic reductions in mean tumor burden when compared with PDT only or C225. Median survival was approximately threefold greater for mice in the PDT + C225 group than for mice in the no-treatment control group [16]. Inhibition of EGFR has been shown to increase the antitumor activity of radiotherapy and chemotherapy in preclinical and clinical studies [49, 50]. In an *in vivo* study on head and neck xenograft model in nude mice, combination of PDT and chemotherapy drug carboplatin reduced the tumor size. Though the difference in the tumor size was not significant between the PDT and combination therapy groups, a difference in the expression of EGFR was observed between these two groups [51]. In our studies we observed that PDT and nimotuzumab synergistically delayed tumor growth. Similarly, del Carmen and colleagues [16] also presented evidence that intraperitoneal administration of C225 (cetuximab) and benzoporphyrin derivative monoacid-A (BPD)-PDT act synergistically to prevent or inhibit tumor cell growth and extend survival in a murine model of ovarian cancer peritoneal metastasis. Another study reported that ERK1/2 and EGFR-PI3K-Akt pathways seem to be involved in cellular survival after PDT and EGFR inhibitors and PDT act synergistically to reduce malignant tumors effectively [52]. Treatment of U87MG brain tumours with nimotuzumab and radiation has also shown enhanced inhibition of EGFR-signalling activation [53]. Such inhibition was not apparent for tumors treated with radiation alone, suggesting a rationale for combined treatment with anti-EGFR mAbs and radiotherapy.

Epidermal growth factor receptor is a receptor tyrosine kinase that regulates important cellular functions including cell cycle progression, cell survival and proliferation, inhibition of apoptosis and induction of angiogenesis. Our results show increased expression of EGFR in PDT treated tumors. The effect of PDT on EGFR remains controversial, some studies have indicated an upregulation of EGFR expression [54] while others have shown degradation of EGFR [13]. The reason for this discrepancy is unclear, but may be due to photosensitizer specific differences, since some of these studies were performed with HPPH and ALA as photosensitizers [55, 56] as opposed to the BPD and porfimer sodium. In an earlier study, we have shown upregulation of EGFR signalling in hypericin mediated PDT of bladder tumors [20]. Though, PDT treated tumors expressed greater EGFR levels, the increase in tumor size was not as significant as the control tumors. This could be due to the fact that various other pro-survival molecules and growth factors could play a role in the proliferation of cancer cells. Downregulation of EGFR was observed in the tumors treated with PDT and nimotuzumab. Immunohistochemical analysis of tumour specimens from head and neck cancer patients treated with the combination of nimotuzumab and radiation also showed evidence of antiproliferative and antiangiogenic effects [57]. It has been reported that the potentiation of the antitumor activity of radiation by nimotuzumab may be related more to the level of EGFR expression at the cell surface rather than to EGFR mutation [43]. EGFR overexpression is associated with tumor progression and poor prognosis in many types of cancers including head and neck cancers [58]. It has been indicated that nimotuzumab has a better clinical effect in tumors that overexpress EGFR. In a trial where 92 treatment-naïve patients with advanced head and neck squamous cell carcinoma received standard therapy either with or without nimotuzumab, EGFR expression showed a significant correlation with patient survival in patients treated with nimotuzumab and chemoradiation [25].

The proliferation marker Ki-67 was high in the control tumors indicating aggressive growth. Ki-67 expression was significantly lower compared to tumors treated with PDT and nimotuzumab only. Nimotuzumab is known to be anti-proliferative and this effect increases when combined with other conventional treatments. It has been demonstrated that nimotuzumab-treated adenoid cystic carcinoma cells were arrested in G1 phase and showed decreased expression of Ki-67 [51]. We observed that the combination of PDT + nimotuzumab decreased CD31 vessel density compared to the control tumors. However, open lumen vasculature was observed in the combination therapy treated tumors which may suggest vessel maturation and increased functionality of vessels. One of the reasons could be the fact that though PDT initially suppressed microvessel density, the effect could have disappeared after a longer period (1 month post PDT). Also open vasculature perhaps could have resulted because of collapse or occlusion in feeding vessels or flow cessation and clot formation [59]. The anticancer properties of nimotuzumab have been associated with potent antiproliferative, antiangiogenic and proapoptotic activity. A431 subcutaneous tumors grown in SCID mice exhibited a reduction in overall microvascular density (MVD) and reduction in Ki-67 positive tumor cell fraction with an elevated apoptotic index [23]. Immunohistochemical studies on biopsies of patients with advanced HNSCC who received nimotuzumab has shown a reduction in proliferation index (*P* = 0.012) and Ki-67 was expression mainly in the infiltrating borders of the tumors [24]. Similar to our results, nimotuzumab in combination with radiotherapy produced a reduction in the size of tumor blood vessels and the number of proliferating cells in subcutaneous tumors [53].

Next, we tested the toxicity of nimotuzumab as it was administered to the animals at a dosage of 10 mg/kg, 3 times a week for a 30 day period. We were interested in understanding the effect of different treatments on the liver and kidneys. The creatinine, urea nitrogen (BUN), asparatate aminotransferase (AST) and alanine aminotransferase (ALT) levels in serum were within the normal ranges and no significant difference was observed between the four different treatment groups. The normal range of these enzymes in female nude mice has been reported in Lu et al. [60]. Though there was mild increase in AST and ALT in the combination therapy group it was not significant. No renal or hepatic toxicity was found in any of the animals and there was no treatment related death. Nimotuzumab has shown encouraging safety profiles in numerous clinical trials [21]. Low adverse event and toxicity rates were observed in phase I clinical trial of Nimotuzumab combined with chemoradiation for esophageal SCC [61]. Nimotuzumab is well tolerated and has lesser skin toxicity as it is known to selectively bind to cells that express moderate to high EGFR levels. Also though nimotuzumab blocks ligand binding, it also allows the receptor to adopt its active conformation, warranting the basal level of signalling needed for the survival of non-tumor cells [42].

In summary, we have shown that nimotuzumab synergistically enhanced the antitumor efficacy of PDT *in vitro* and *in vivo*. Our data suggest that the anti-tumor activity of this combination therapy involves the reduced expression of EGFR, Ki-67 and CD31, therefore demonstrating anti-proliferative and anti-antiangiogenic effects with low toxicity. This provides a rationale for future clinical investigations of the therapeutic efficacy of nimotuzumab in combination with PDT.

## MATERIALS AND METHODS

### Cell lines, culture, photosensitizer and inhibitors

Oral squamous carcinoma cell line (OSCC, CAL-27), human squamous carcinoma cell line (HSC-3), squamous cell carcinoma (SCC-25) and human umbilical vein endothelial cells (HUVECs) were obtained from American Type Culture Collection (ATCC, USA). The cells were cultured as a monolayer in RPMI-1640 medium supplemented with 10% fetal bovine serum (Gibco, USA), 1% non-essential amino acids (Gibco, USA), 1% sodium pyruvate (Gibco, USA), 100 units/ml penicillin/streptomycin (Gibco, USA) and incubated at 37°C, 95% humidity and 5% CO_2_. The photosensitizer, Chlorin e6 was provided by ApoCare GmBH, Germany and the EGFR inhibitor nimotuzumab was provided by Innogene Kalbiotech Pte Ltd, Singapore. Erbitux (cetuximab) and Avastin (bevacizumab) were purchased from Imclone Systems and Genentech USA.

### Immunofluorescence

OSCC, HSC-3, SCC-25, human epithelial carcinoma cell line (A431) and human breast adenocarcinoma cell line (MCF-7) cells were seeded at 25,000 cells per well in 8 well chamber slides (Nunc Lab-Tek II chamber slides, Thermo scientific, USA) and were allowed to grow overnight at 37°C. A431 was used as the positive control and MCF-7 was the negative control. The cells were rinsed with PBS and blocking was performed with 1% BSA in PBS for 1 h at 37°C. After removing the blocking buffer the cells were incubated with 1:50 EGFR primary antibody (Rabbit mAb, CellSignaling Technology, USA). After 1 hour incubation the chamber slides were washed three times with PBS and incubated with 1: 100 secondary antibody (Goat polyclonal H&L (TR), Abcam). The slides were then washed 3 times. Hoechst 33342 (PromoCellGmBH, Germany) was used to stain the nucleus. After washing, vectashield mounting medium was used to mount the chamber slides. A fluorescence microscope (Nikon Eclipse 80i, USA) was used to capture the images. The fluorescence intensity was quantified using Image Pro Plus 6.0 software.

### Western blot analysis

OSCC, HSC-3, SCC-25, A431 and MCF-7 cells were lysed in mammalian lysate buffer (M-PER, Pierce, IL, USA) with protease inhibitor (Complete Mini, Roche, Germany). Fifty micrograms of total protein from each cell lysate samples were then resolved on a 10% bis-tris gel before transferring to a nitrocellulose membrane. The membrane was then probed with 1:200 anti-EGFR monoclonal antibody (Cell Signaling Technology, USA) and 1:400 horseradish peroxidase-conjugated antibody (Cell Signaling Technology, USA) using the iBind Western System (Life Technologies, Carlsbad, USA). The membrane was then incubated with chemiluminescent substrate (Thermo Fisher Scientific, USA) followed by detection on traditional ECL film (Amersham Hyperfilm ECL, GE Healthcare, USA). The intensity of the bands was quantified using NIH Image J (1.41o, W. Rasband, National Institute of Health, USA) software.

### Chemotaxis cell migration assay

QCM chemotaxis cell migration assay (Merck Millipore, USA) was used to perform this experiment. 150 μL of cell culture medium with 10% fetal bovine serum was added to the wells of the feeder tray (lower chamber) in a 96 well plate. OSCC, HSC-3, SCC-25 and human umbilical vein endothelial cells (HUVEC) cells were seeded at 5 × 10^4^ cells in 100 μl/well to the cell culture insert. To these wells either bevacizumab, nimotuzumab and cetuximab were added at two different concentrations of 50 μg/ml and 100 μg/ml and human recombinant vascular endothelial growth factor (VEGF) and EGF protein were added at a concentration of 50 ng/ml and 100 ng/ml. The plates were then incubated for 24 hours at 37°C in a CO_2_ incubator. The 8 mm pore-size of the membrane ensured the minimization of non-specific and random migration. Following incubation, the medium was discarded from the top side of the insert by flipping out the remaining cell suspension, and the migration chamber plate was placed onto a new 96-well feeder tray containing 150 μL of pre-warmed cell detachment solution in the wells. 30 minutes after incubation, the cells were allowed to dislodge completely from the underside by gently tilting the migration chamber plate back and forth several times during incubation. Next, 50 μL of lysis buffer/calcein AM solution was added to each well of the feeder tray containing 150 μL cell detachment solution with the cells that migrated through the membrane. The plate was then allowed to incubate for 15 minutes at room temperature. 150 μL of this mixture was transfered to a new 96-well plate that was suitable for fluorescence measurement. A fluorescence plate reader (Glomax multi detection system, Promega USA) using 480/520 nm filter set was used to perform the measurements.

### Extracellular matrix cell invasion assay

QCM ECMatrix cell invasion assay, 96-well (Merck Millipore, USA) was used to perform this assay. 150 μL of cell culture medium with 10% fetal bovine serum was added to the wells of the feeder tray (lower chamber) in a 96 well plate. OSCC, HSC-3, SCC-25 and HUVEC cells were seeded at 5 × 10^4^ cells in 100 μl/well to the cell culture insert. To these wells either bevacizumab, nimotuzumab and cetuximab were added at two different concentrations of 50 μg/ml and 100 μg/ml and VEGF and EGF protein were added at a concentration of 50 ng/ml and 100 ng/ml. The plates were then incubated for 24 hours at 37°C in a CO_2_ incubator to permit invasion. Following incubation, the medium was discarded from the top side of the insert by flipping out the remaining cell suspension, and the migration chamber plate was placed onto a new 96-well feeder tray containing 150 μL of pre-warmed cell detachment solution in the wells. 30 minutes after incubation, the cells were allowed to dislodge completely from the underside by gently tilting the migration chamber plate back and forth several times during incubation. Next, 50 μL of lysis buffer/calcein AM solution was added to each well of the feeder tray containing 150 μL cell detachment solution with the cells that migrated through the membrane. The plate was then allowed to incubate for 15 minutes at room temperature. 150 μL of this mixture was transferred to a new 96-well plate that was suitable for fluorescence measurement. A fluorescence plate reader (Glomax multi detection system, Promega USA) using 480/520 nm filter set was used to perform the measurements.

### Endothelial tube formation assay

Endothelial cell tube formation assay (BD BioCoat™ Angiogenesis System, NJ, USA) was used to perform this experiment. The 98-well plate coated with matrigel was thawed overnight at 4°C and the matrigel matrix was allowed to polymerize for 30 minutes at 37°C and 5% CO_2_ environment. 2 × 10^4^ HUVEC cells in 50 μl of medium was added to each well. At the same time EGFR inhibitors nimotuzumab (50 μg/ml) and cetuximab (50 μg/ml) were added to the medium. Angiogenesis promoter VEGF (50 ng/ml) and negative control sulphophorane (100 μM) were added separately to the cells. The plate was incubated for 16 to 18 hours at 37°C, 5% CO_2_ atmosphere. Following incubation, medium was carefully removed from the plates and the cells were labelled with calcein AM. After 40 min incubation, the cells were washed twice and tube formation of HUVEC cells was captured using a fluorescence microscope (Zeiss, Axiovert 200M). The length of the tubes was measured using Image Pro Plus 6.0 software.

### Cell viability assay

CellTiter-Glo^®^ luminescent cell viability assay (Promega, USA) was used to perform the cell viability assay. It is a homogeneous method to determine the number of viable cells in culture based on quantitation of the ATP present, which signals the presence of metabolically active cells. OSCC HSC-3, SCC-25 and HUVEC cells were seeded in 96-well (10,000 cells per well) opaque-walled plates. Control wells containing medium only were used to subtract background luminescence. After overnight incubation, the cells in the wells were treated with the following combinations of treatment in triplicates: (i) control (cells only), (ii) Chlorin e6 – 50 μM and 100 μM, (iii) Nimotuzumab – 50 μg/ml and 100 μg/ml, (iv) Cetuximab – 50 μg/ml and 100 μg/ml, (v) Light irradiation alone 1 J/cm^2^, (vi) PDT at 1 J/cm^2^ with Chlorin e6 concentrations of 50 μM and 100 μM (vii) Combination of PDT and nimotuzumab (nimo) at four different dosages for e.g. (a) PDT (50 μM)/nimotuzumab (50 μg/ml), (b) PDT (50 μM)/nimotuzumab (100 μg/ml), (c) PDT (100 μM)/nimotuzumab (50 μg/ml) and (d) PDT (100 μM)/nimotuzumab (100 μg/ml), (vii) Combination of PDT and cetuximab at four different dosages for e.g. (a) PDT (50 μM)/cetuximab (50 μg/ml), (b) PDT (50 μM)/cetuximab (100 μg/ml), (c) PDT (100 μM)/cetuximab (50 μg/ml) and (d) PDT (100 μM)/cetuximab (100 μg/ml). Treatment was performed using two 96 well plates. For the control plate Ce6 alone, nimotuzumab and cetuximab alone was added to the cells and incubated for 24 hours. The cells in the treatment plate were treated with light only, PDT and the combination therapies of PDT + nimotuzumab and PDT + cetuximab. For PDT treatment, chlorin e6 was added to the medium with cells and incubated for 3 hours. Before light irradiation, the cells were rinsed with PBS and chlorin e6 was removed. Fresh medium was added and PDT was performed. For the combination therapy groups, after PDT the medium was removed and fresh medium with nimotuzumab and cetuximab was added. 24 hours after treatment 100 μl of CellTiter-Glo™ Reagent was added to 100 μl of medium present in each well. The contents were mixed for 2 minutes on an orbital shaker to induce cell lysis. The plate was then allowed to incubate at room temperature for 10 minutes to stabilize luminescence signal. Luminescence was recorder with a plate reader (Glomax multi detection system, Promega USA).

### *In vivo* treatment protocol

Oral squamous carcinoma cells were cultured, harvested and suspended in Hank's balanced salt solution (HBSS) (Gibco, USA). A cell suspension of 3 × 10^6^ was prepared in 100 μl of HBSS and this was injected subcutaneously into the flanks of 6–8 weeks Balb/c nude mice. When the tumors reached 200 mm^3^ in diameter, the animals were randomly assigned to 4 groups (10 animals per group) for tumor regression and survival experiments, (i) Control (mice with untreated tumors), (ii) PDT only, (iii) nimotuzumab only and (iv) PDT + nimotuzumab. PDT treatment involved the intravenous injection of 10 mg/kg chlorin e6, 3 hours later the tumor was irradiated with a laser light source with a wavelength of 665 nm (LVI Technology Inc, South Korea). A light dosage of 150 J/cm^2^ and fluence rate of 100 mW/cm^2^ was used for PDT treatment. The animals were anesthetized with isoflurane during PDT. Nimotuzumab was administered intraperitonially (10 mg/kg) at time 0 (immediately after light exposure), 24 h, 48 h and then 3 times a week for up to 32 days post PDT for tumor regression experiments. The tumor size was estimated using the formula, volume = (π/6 × d1 × d2 × d3), where d1, d2 and d3 are tumor dimensions in 3 orthogonal directions. For survival studies, the animals were monitored for 90 days. Mice were euthanized when either the tumor reached the 2 cm^3^ ethical limit or at the end of the 90-day monitoring period. All procedures were approved by the Institutional Animal Care and Use Committee (IACUC), Singapore Health Services Pte Ltd, and performed in accordance with international standards.

### Statistical calculation of PDT response for *in vitro* and *in vivo* treatment

A statistical model was used to assess synergistic, additive and antagonistic effects of the combination therapy based on Weyergang et al. [28]. Briefly, it is assumed that the mechanistic action of PDT and angiogenesis inhibitors can be distinct. The additive (add) effect of two treatments is the product of the survival fraction (SF) of each treatment which is SF_add_ = SF_PDT_ × SF_Nimo_. The SFs were calculated by dividing the number of treated cells by the average number of untreated cells in 3 independent experiments. The calculated SF_add_ was then compared to the observed SF_comb_, which are the PDT + nimotuzumab combination therapy groups. The calculated SF_add_ was then compared to the observed SF_comb_ by the synergy/antagonism parameter difference in logarithms (DL), defined as the difference in logarithms between the observed SF_comb_ and the calculated SF_add_.

$$DL=\log{\text{SF}}_{\text{PDT}}+\log{\text{SF}}_{\text{Nimo}}-\log{\text{SF}}_{\text{comb}};$$

Synergistic effects will result in positive DL values; negative DL values will indicate antagonism while DL equal to zero will indicate additive effect of the treatments. Significant deviations of DL from zero were established through *t*-tests based on a two-tailed significance level of *p* = 0.05.

### Detection of EGFR, Ki-67 and CD31 using immunohistochemistry

Processing of the samples was done using tissue processor (Leica TP 1020, Germany). Briefly the tissue samples were fixed in 10% formalin for 24 h, and then processed in an ascending series of ethanol and subsequently cleared with xylene and embedded in paraffin. The paraffin embedded samples of oral cancer xenograft tumors were cut at a thickness of 4 μm using a microtome (Leica RM 2135, Germany). The sections were mounted on superfrost/plus slides (Fischer Scientific, USA) and air-dried. On the day of staining the slides, the parafilm was cleared in Neo-clear (Merck Millipore, USA) twice for 10 min before rehydrating in ethanol series. Sections were incubated with hydrogen peroxide for 10 min to block endogenous peroxidase activity. After which, the sections were incubated overnight at 4°C with primary antibodies, i.e., EGFR and Ki-67 (1:100; Cell Signaling, USA). For CD31 staining, chemicon blood vessel staining kit (Millipore, USA) was used. To confirm the specificity of binding, normal mouse serum IgG_1_ (1:500) was used as a negative control. Following extensive washing, sections were incubated for 30 min in the secondary biotinylated antibody, rinsed and followed by DAB Chromogen (Vector Laboratories, UK) for 10 min. Sections were then counter-stained with Harris's hematoxylin and dehydrated in ascending grades of ethanol before clearing in xylene and mounting under a cover slip. Images were captured using imaging software (NIS Elements, Nikon). The images were saved in TIFF format and NIH Image J (1.41o, W. Rasband, National Institute of Health, USA) software was used to analyze and quantify the expression of EGFR, Ki-67 and CD31. For EGFR and Ki-67, the percentage of staining was calculated. For CD31, microvessel density (MVD) was quantified per mm^2^ area.

### Assessment of toxicity

We assessed the kidney function of the animals post treatment. Urea nitrogen (BUN) and serum creatinine colorimetric kit (Arbor assay, USA) was used to measure urea and creatinine levels in serum to assess kidney toxicity. Briefly, for urea nitrogen, 50 μL of serum was added to a 96 well plate. 50 μL of water was added into duplicate wells as the Zero standard. 75 μL of Color Reagent A followed by 75 μL of Color Reagent B was added to each well. This was then incubated at room temperature for 30 minutes. The optical density was read at 450 nm.

For serum creatinine assay, 25 μL of mouse serum was added to a 96 well plate. Water was used as blank and standards were also included. 25 μL of Assay diluent was later added to all the wells. After which, 100 μL of the DetectX^®^ Creatinine Reagent was added to each well. After 1 min incubation at room temperature, the plate was read at 490 nm. Again after 30 minutes, the optical density was recorded at 490 nm. The average optical density of the standards at 1 minute was subtracted from the average optical density of the standards at 30 minutes and the results were plotted against the creatinine concentration of the standards. The concentration of unknown samples was calculated based on the linear regression line generated.

For the Aspartate Aminotransferase (AST) assay, 5 μl of serum was added to the assay diluent in a 96 well plate. 100 μl reactive mix consisting of AST Assay Buffer, AST Enzyme Mix, Developer and AST Substrate was added to each well containing the standards and samples. For Alanine Aminotransferase (ALT) assay, 100 μl of reactive mix was prepared by mixing ALT Assay Buffer 86 μl, OxiRed Probe 2 μl, ALT Enzyme Mix 2 μl and ALT Substrate 10 μl. 100 μl of the reaction mix was added to each well containing the serum samples and standards. The optical density was read at 10 min and 60 min post incubation.

AST and ALT activity in the test samples was then be calculated by:

AST/ALT Activity=B/T2-T1*V=nmol/min/ml=mU/ml

where B is the pyruvate amount from pyruvate standard curve (in nmol). T1 is the time of the first reading (in min). T2 is the time of the second reading (in min). V is the original sample volume added into the reaction well (in ml).

### Statistical analysis

Two-way analysis of variance (ANOVA) with Tukey's multiple comparisons was performed to analyze the tumor regression data using Prism 6.0 software (Graphpad Prism, San Diego, CA). For all *in vitro* studies ordinary one-way ANOVA with Tukey's multiple comparisons was performed. A *p* value of < 0.05 was considered to be significant. For synergistic measurements, significant deviations of DL from zero were established through *t*-tests based on a two-tailed significance level of *p* = 0.05.

## SUPPLEMENTARY FIGURES


