# Supplementary material for: Nimotuzumab increases the anti-tumor effect of photodynamic therapy in an oral tumor model
Source: Oncotarget. 2015 Apr 20;6(15):13487–505. doi: 10.18632/oncotarget.3622 (PMC4537029; doi:10.18632/oncotarget.3622)
Supplement: Supplementary file 1 [file oncotarget-06-13487-s001.pdf]

SUPPLEMENTARY FIGURES

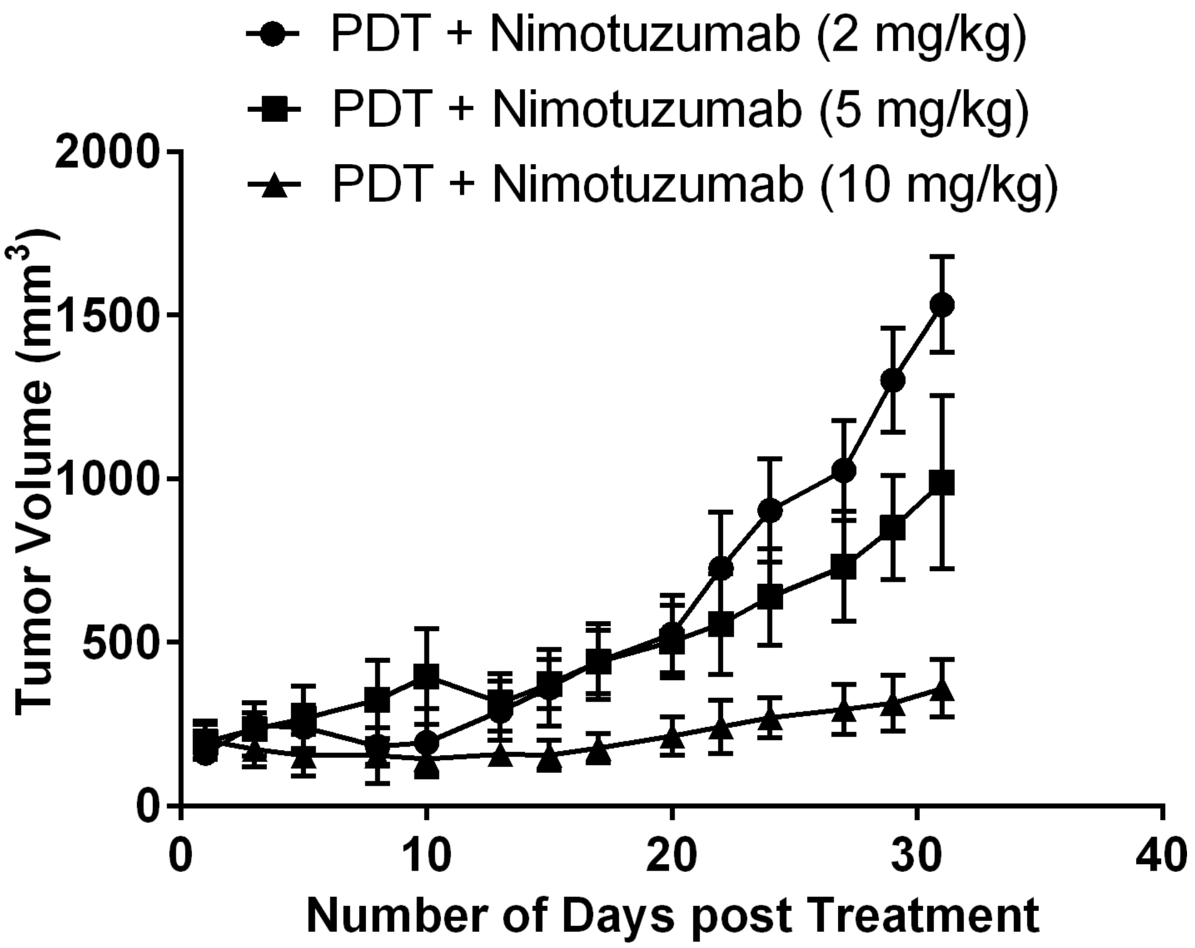

Supplementary Figure 1: Tumor volume was charted against days, to assess the tumor response in various treatment groups for 30 days. Each group represents the mean (bars, SE) of 5 animals.

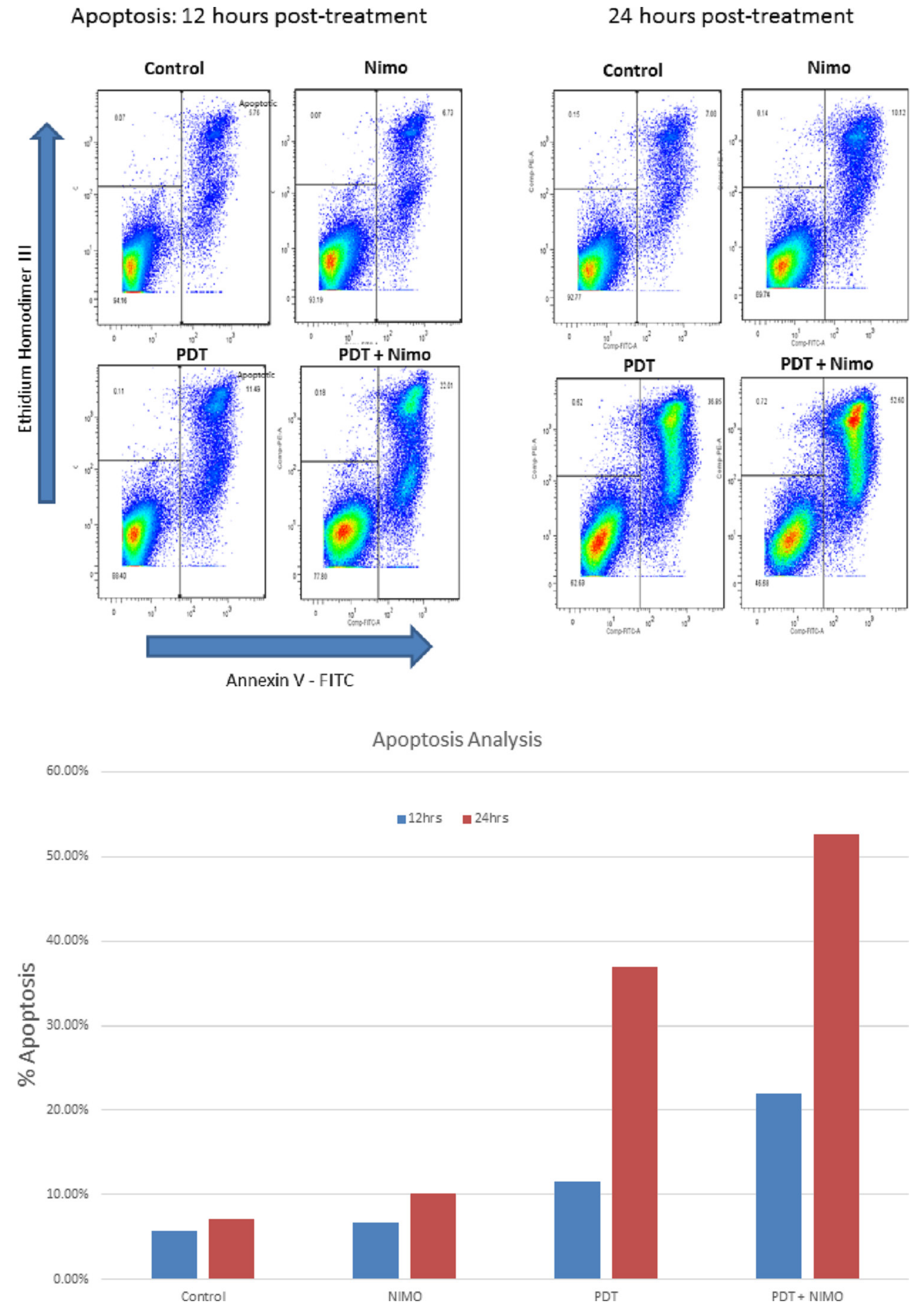

Supplementary Figure 2: Apoptotic cell death after 12 and 24 hrs post treatment was determined using flow cytometry.
